# Radiation-Inactivated *S*. *gallinarum* Vaccine Provides a High Protective Immune Response by Activating Both Humoral and Cellular Immunity

**DOI:** 10.3389/fimmu.2021.717556

**Published:** 2021-08-16

**Authors:** Hyun Jung Ji, Eui-Baek Byun, Fengjia Chen, Ki Bum Ahn, Ho Kyoung Jung, Seung Hyun Han, Jae Hyang Lim, Yongkwan Won, Ja Young Moon, Jin Hur, Ho Seong Seo

**Affiliations:** ^1^Research Division for Radiation Science, Korea Atomic Energy Research Institute, Jeongeup, South Korea; ^2^Department of Oral Microbiology and Immunology, and DRI, School of Dentistry, Seoul National University, Seoul, South Korea; ^3^Research and Development Center, HONGCHEON CTCVAC Co., Ltd., Hongcheon, South Korea; ^4^Department of Microbiology, Ewha Womans University College of Medicine, Seoul, South Korea; ^5^Ewha Education & Research Center for Infection, Ewha Womans University Medical Center, Seoul, South Korea; ^6^Department of Veterinary Public Health, College of Veterinary Medicine, Jeonbuk National University, Iksan, South Korea; ^7^Department of Radiation Science, University of Science and Technology, Daejeon, South Korea

**Keywords:** salmonellosis, inactivated vaccine, radiation inactivation, IgG2b, IgG3, CD4^+^ T cells, fowl typhoid

## Abstract

*Salmonella enterica* subsp. *enterica* serovar Gallinarum (SG) is a common pathogen in chickens, and causes an acute systemic disease that leads to high mortality. The live attenuated vaccine 9R is able to successfully protect chickens older than six weeks by activating a robust cell-mediated immune response, but its safety and efficacy in young chickens remains controversial. An inactivated SG vaccine is being used as an alternative, but because of its low cellular immune response, it cannot be used as a replacement for live attenuated 9R vaccine. In this study, we employed gamma irradiation instead of formalin as an inactivation method to increase the efficacy of the inactivated SG vaccine. Humoral, cellular, and protective immune responses were compared in both mouse and chicken models. The radiation-inactivated SG vaccine (r-SG) induced production of significantly higher levels of IgG2b and IgG3 antibodies than the formalin-inactivated vaccine (f-SG), and provided a homogeneous functional antibody response against group D, but not group B Salmonella. Moreover, we found that r-SG vaccination could provide a higher protective immune response than f-SG by inducing higher Th17 activation. These results indicate that r-SG can provide a protective immune response similar to the live attenuated 9R vaccine by activating a higher humoral immunity and a lower, but still protective, cellular immune response. Therefore, we expect that the radiation inactivation method might substitute for the 9R vaccine with little or no side effects in chickens younger than six weeks.

## Introduction

Salmonellosis is a zoonotic disease that can cause gastroenteritis, diarrhea, and systemic typhoid in humans and animals. *Salmonella* species are gram-negative, facultatively flagellated bacteria classified on the basis of 46 lipopolysaccharide (LPS, O) and 114 flagella (H) antigens (1). According to this taxonomy, more than 2,610 serotypes have been identified to date (2). Among them, *Salmonella enterica* subsp. *enterica* serovar Gallinarum (*S.* Gallinarum; SG) is known to cause invasive salmonellosis, or fowl typhoid-like disease, a septic disease that occurs in both acute and chronic forms in chickens, turkeys, and other birds (3). Although SG infection has largely disappeared in the poultry industry in developed countries, it is still widespread in developing countries, causing enormous annual economic losses (4).

Vaccination of chickens has provided promising protection, and there continues to be progress in the development of a safe and efficacious Salmonella vaccine that provides broad cross-protection for enhancing both animal health and food safety (5). The most commonly used vaccine is a commercial live vaccine derived from a stable rough strain of SG 9R that was developed more than 30 years ago (6). Although the protective efficacy of this vaccine has been reported to be extremely high (7), the remaining pathogenicity in this attenuated strain can also lead to severe systemic infections in immunosuppressive groups such as young chicks (8). In addition, the problem of pathogenic reversion due to natural mutation in this strain has also been reported, leading to an increasing demand for additional vaccine development (9). In fact, SG 9R strains from three different Korean animal vaccine companies show different phenotypic characteristics and vaccine efficacy despite having the same original strain (10).

An inactivated vaccine can be considered as a safer alternative to SG 9R. However, several reports have shown that inactivated SG vaccines are not sufficient to provide protection against salmonellosis and less cross-protective against other Salmonella species, such as *Salmonella* Pullorum (SP) or *Salmonella* Enteritidis (SE) because of the low cell-mediated immune response (11, 12). Of the several inactivation methods available for vaccine development, inactivation by irradiation has been reported to enhance the induction of cell-mediated immunity for bacterial and viral vaccines (13, 14). Radiation, such as gamma and X-rays, transfers energy to produce ionization that directly or indirectly damages dsDNA (15). This ionization is completed in picoseconds (~10^–12^ s), so it is thought that it will cause less immunogenic damage that could induce cellular immunity. The major advantages of ionizing radiation in vaccine development, compared to formalin, are the ability to penetrate most biological materials, and the fact that it targets both double- and single-stranded nucleic acids while causing less damage to antigenic surface proteins. Moreover, there is no need to remove any chemical residue after inactivation  (16). Gamma-irradiated influenza vaccine was more effective at priming cross-reactive cytotoxic T cells and protected mice against a heterologous influenza virus (17). Irradiated bacterial vaccines, such as against Listeria, Mycobacteria, and Bacillus, which prevent replication but retain their metabolic activity, generate higher cell-mediated immune responses and protect against extracellular and intracellular bacteria (14, 18–20).

In this study, we prepared an SG vaccine using gamma irradiation and analyzed its efficacy by measuring the immune response in mice and chickens. This study demonstrates that gamma irradiation is suitable for developing inactivated vaccines against SG and other infectious diseases.

## Materials and Methods

### Ethics Statement

This study was performed in strict accordance with the recommendations of the Guide for the Care and Use of Laboratory Animals of the National Institutes of Health. All animal experiments were approved by the Committee on the Use and Care of Animals at the Korea Atomic Energy Research Institute (KAERI; approval no. IACUC-2018-007) and performed according to accepted veterinary standards set by the KAERI animal care center. Mice were euthanized by CO_2_ inhalation, as specified by the KAERI Institutional Animal Care and Use Committee guidelines.

### Bacterial Strains

*S.* Gallinarum 07Q015 was obtained from the Korea Veterinary Culture Collection (Kimchun, Republic of Korea), and its genome was sequenced using the PacBio RS II platform (Pacific Biosciences, Menlo Park, CA, USA) at Macrogen Co., Ltd. (Seoul, Republic of Korea). The assembled genome of *S.* Gallinarum 07Q015 contained three contigs, one circular genome (4,624,135 bp) and two plasmids (88,418 bp and 56,404 bp). After complete genome assembly, BLAST analysis (v2.7.1) was carried out to identify the species to which each scaffold showed the highest similarity. The best hit was *S. enterica* subsp. *enterica* serovar Gallinarum strain 287/91 (accession number: AM933173.1). The whole genome sequence of *S.* Gallinarum 07Q015 has been deposited at DDBJ/EMBL/GenBank under accession no. CP077760 (Contig 1), CP077761 (Contig 2), CP077762 (Contig 3). In the challenge experiment, we assessed the median lethal dose (LD_50_) and the optimal challenge dose by monitoring the survival of mice and chicken for two weeks after *S.* Gallinarum 07Q015 inoculation intraperitoneally (i.p.) and orally, respectively. The LD_50_ was calculated using the Reed–Muench method. The optimal challenge doses were at least 10 times higher than LD_50_, i.e., 5 × 10^5^ CFU for mice and 3 × 10^7^ CFU for chicken.

### Preparation of Radiation- or Formalin-Inactivated SG (r-SG or f-SG)

Salmonella was grown in Luria-Bertani (LB; Difco, BD Biosciences, Franklin Lakes, NJ, USA) broth at 37°C and 200 rpm under aerobic conditions. When the bacteria culture reached an optical density (OD_600_) of 0.8, it was pelleted by centrifugation at 7000 rpm for 20 min at 4°C and resuspended in phosphate-buffered saline (PBS). Harvested SG (10^8^–10^9^ CFU/mL) was irradiated using a ^60^Co-gamma irradiator (point source AECL, IR-79, MDS Nordion International Co., Ottawa, Canada) at the Advanced Radiation Technology Institute of KAERI (Jeongeup, Republic of Korea) with an absorbed dose of 0.5–9 kGy for 1 h at 23°C. f-SG was prepared by incubation with 0.2% formaldehyde solution (JUNSEI; Tokyo, Japan) under mild agitation at 23 - 28°C for 2 h. To confirm inactivation of the prepared vaccines, samples were inoculated in LB broth and cultured for 3 d to assess SG growth.

### Quantification of DNA Damage

Genomic DNA was extracted using a G-spin™ Genomic DNA extraction Kit (INTRON Inc., Seoul, Republic of Korea). The genomic DNA concentration was measured using a spectrophotometer (Epoch 2; BioTek, Winooski, VT, USA) and titrated to 100 μg/mL in 10mM Tris-EDTA buffer. To quantify single-strand DNA breaks, a DNA Damage Quantification Kit (Dojindo Laboratories, Kumamoto, Japan) was used according to the manufacturer’s instructions. Briefly, genomic DNA (1 µg) was labeled with an aldehyde reactive probe conjugated with biotin, through incubation at 37°C for 1 h. Labeled DNA was immobilized on a 96-well U-bottom microplate (Dojindo) and incubated at 23°C overnight. Bound biotin-conjugated aldehyde reactive probe on immobilized genomic DNA was detected using peroxidase-streptavidin and the OD of the conjugated aldehyde reactive probe was measured at 650 nm after adding 100 µL of 3,3’,5,5’-tetramethylbenzidine substrate solution.

### Measurement of Protein Carbonylation

SG samples were sonicated using a TECAN sonicator (TECAN; Osaka, Japan) for 1 min on ice. The samples were then centrifuged at 10,000 × g for 15 min at 4°C. Protein concentration and purity were measured using absorbance at 280 nm and 260 nm, respectively. Protein carbonylation was measured using a Protein Carbonyl Colorimetric Assay Kit (Cayman Chemical, Ann Arbor, Michigan, USA) according to the manufacturer’s instructions. In brief, 200 µL of the sample was mixed with 800 µL of 2,4-dinitrophenylhydrazine and 800 µL of 2.5 M HCl. After the sample was incubated for 1 h at room temperature, the proteins were precipitated using 20% trichloroacetic acid at 4°C for 5 min. After centrifugation, the pellet was washed with 1 mL of a mixture of ethanol and ethyl acetate (1:1, v/v) and resuspended with protein pellets in 500 µL of guanidine hydrochloride. The OD of the pellet was measured at 360–385 nm.

### Scanning Electron Microscope (SEM) Analysis

Live SG, r-SG, and f-SG were fixed with 4% glutaraldehyde overnight at 4°C. After centrifugation (13,000 rpm), the samples were washed three times with PBS and dehydrated through a graded ethanol series (30%, 50%, and 70%) followed by drying the cells. The samples were gold-coated using a gold sputtering unit and then observed using a JEOL JSM-840 scanning microscope (Tokyo, Japan) at the Seoul National University.

### Mouse and Chicken Experiments

The animal housing conditions, which were designed for specific pathogen-free animals, and the animal experimental design were approved by the Committee on the Use and Care of Animals at the KAERI and implemented ethically according to the standards accepted by the National Health of Institute. The ventilated housing cage (Orient Bio Inc., Seoul, Republic of Korea) was maintained in an animal biological safety level 2 facility at 22–23°C on a 12 h:12 h light:dark cycle. The cages were covered with high-efficiency particulate air-filtered microisolation lids (Orient Bio Inc.) in a static airflow environment. Bedding (Aspen shavings; Orient Bio Inc.) at an approximate depth of 1.0 cm was changed weekly. Irradiated rodent diet food and sterile water were provided *ad libitum* through a wire cage top. Five-week-old male C57BL/6 mice (weight 19–21 g) were purchased from Orient Bio Inc. Five C57BL/6 mice were randomly assigned to individually ventilated housing cages and immunized i.p. three times at two-week intervals with either PBS, r-SG, or f-SG mixed with the same volume of 2% Alhydrogel^®^ adjuvant (InvivoGen, San Diego, CA, USA). No significant weight loss, mortality, or serious clinical signs were observed after vaccination. Two weeks after the third vaccination, blood was collected to measure SG-specific antibodies, and the spleen was collected to measure SG-specific T cell responses. To examine the protective efficacy of the vaccination, mice were challenged i.p. with *S.* Gallinarum 07Q015 (5 × 10^5^ CFU/mouse) two weeks after the third vaccination. Mouse survival was monitored for 12 d.

Five-week-old female Brown Leghorn chickens (weight 5-6 lb) were purchased from JOIN Inc. (Pyeongtaek, Korea) and placed into chicken isolator (Three-shine Inc.; Daejeong, Korea) containing mesh wire on the floor of the cages. Each cage housed not more than 5 chickens. Chickens were given tap water and commercial chick feed *ad libitum*. Air and light were supplied freely through the hole and windows. All purchased chickens were confirmed to be negative for *Salmonella* infection by confirming with *Salmonella* diagnosis PCR with Primers 3503 (AGC GTA CTG GAA AGG AG) and 5503 (ATA CCG CCA ATA AAG TTC ACA AAG) (21). Chickens adapt to the new environment for one week. All chickens were acclimatized according to the protocols of the Central Animal Research Laboratory at Chunbuk National University (Iksan, Republic of Korea). For vaccination, chickens (n = 10/group) were vaccinated into the breast muscle twice at three-week intervals with r-SG (3 × 10^8^ CFU), f-SG (3 × 10^8^ CFU), or live 9R strain (2 × 10^7^ CFU) of 0.5 mL. Three weeks after the second vaccination, chicken blood was collected 5 mL each from brachial wing vein, and serum was harvested from the supernatant of coagulated blood. All serum were stored at 4°C until performing enzyme-linked immunosorbent assay (ELISA) and opsonophagocytic killing assay (OPKA). For *Salmonella* challenge study, *S.* Gallinarum 07Q015 were inoculated into 10 mL LB and incubated at 37°C with orbital shaking (200 rpm) for 24 h. Subsequently, 0.5 mL of precultures bacteria was transferred into 50 mL LB and incubated with orbital shaking (200 rpm) at 37°C for about 6 h. The challenge inoculum was prepared by diluting *S.* Gallinarum culture in LB to a final viable concentration of 6 × 10^7^ CFU/mL and used immediately for oral infection. The number of *S*. Gallinarum inoculums was determined both before and after challenge. Three weeks after the second vaccination, chickens were orally challenged with 0.5 mL of *S.* Gallinarum 07Q015 (3 × 10^7^ CFU/chicken) as inoculum using a syringe, whereas uninfected chickens were given with the same amount of LB. Chicken survival was monitored daily for 15 d. Any chicken that died or had to be euthanized during the observation period was immediately necropsied and all the remaining chickens at the end of the experiment were euthanized.

### Measurement of Lipopolysaccharide (LPS)-Specific Immunoglobulin Levels

Blood samples from mice and chickens were obtained 14 d after the last vaccination. Salmonella antigen lysates were prepared as described previously (22). *Salmonella* Gallinarum (SG), *Salmonella* Typhimurium (ST), *Salmonella* Enteritidis (SE), and *Salmonella* Pullorum (SP) were grown in LB broth and harvested at OD_600_ = 0.8. The pellet was washed with PBS followed by sonication 30 times for 5 s. Samples were centrifuged at 13,000 rpm for 10 min at 4°C, and the supernatants were collected and stored at –70°C. Total protein concentration was measured using the Pierce™ BCA Protein Assay Kit (Thermo Fisher Scientific, Waltham, MA, USA). To examine the levels of SG-specific immunoglobulins (Igs), Salmonella lysate (10 μg/well), group D LPS (1 μg/well; Sigma-Aldrich; St. Louis, MO), and group B LPS (1 μg/well; Sigma-Aldrich) were immobilized on 96-well plates for 16 h at 4°C, followed by blocking with 1% BSA in PBS. After washing three times with PBS containing 0.05% Tween-20 (PBS-T; Sigma-Aldrich), serial two-fold dilutions of mouse or chicken serum (100 μL) were added to each well and incubated at 23°C for 2 h. The plates were washed five times with PBS-T to remove unbound antibodies, and bound antibodies were detected using horseradish peroxidase (HRP)-conjugated anti-mouse Igs (anti-mouse IgM, IgG, IgG1, IgG2a, IgG2b, and IgG3; 1:5000 dilution in PBS-T; Sigma-Aldrich) or HRP-conjugated anti-chicken IgM and IgG (1:5000 dilution in PBS-T; Southern Biotech, Birmingham, AL, USA) for 1 h at room temperature. After washing seven times with PBS-T, 100 μL of 3,3’,5,5’-tetramethylbenzidine substrate solution (INTRON) was added, followed by incubation for 5–10 min at 23°C. When the color was sufficiently developed, 50 μL of 2 N H_2_SO_4_ stop solution (Daejung Chemicals; Siheung, Republic of Korea) was added. The absorbance at 450 nm was measured using an Epoch 2 plate reader (BioTek).

### Opsonophagocytic Killing Assay (OPKA)

The functional activity of SG-specific antibodies induced by each vaccine was assessed using OPKA as described previously (23). A 10 μL aliquot of bacteria (100–250 CFU) was incubated with three-fold serially diluted heat-inactivated sera (final 20 μL/well) with OBB (Opsonization buffer B, 1X HBSS buffer containing 0.1% gelatin and 0.5% heat inactivated FBS) at 37°C for 30 min, followed by mixing with 40 μL of differentiated HL60 granulocytic cells (1 × 10^7^ cells; ATCC; Manassas, VA, USA) and 10 μL of 3–4-week-old baby rabbit complement (PelFreeze Biologicals, Rogers, AR, USA). After incubating at 37°C for 45 min, the reaction was stopped by incubating on ice for 20 min. Next, 10 μL of samples were spotted on LB agar plates and the colonies were counted using NIST’s Integrated Colony Enumerator software (NICE; Gaithersburg, MD, USA) and opsonic indices (OIs) were analyzed using the Opsonization Index Program (“opsotiter3”) kindly provided by Prof. Nahm (University of Alabama at Birmingham).

### Splenocyte Analysis by Flow Cytometry

Two weeks after the final immunization, spleens from mice immunized with either the r-SG or f-SG vaccine were isolated and filtered through a cell strainer (70 µm; SPL Life Sciences, Pocheon, Republic of Korea). Red blood cells (RBCs) were lysed with RBC lysis buffer (Sigma-Aldrich) and washed with RPMI-1640 medium containing 10% fetal bovine serum (FBS; Biowest, Nuaillé, France). The cell suspension was seeded into a 48-well plate (2 × 10^6^ cells/well) and stimulated with 10 µg/mL SG lysate, 0.5 µg/mL GolgiStop (BD Bioscience, San Diego, CA, USA), and 0.5 µg/mL GolgiPlug (BD Bioscience) at 37°C for 12 h. The cells were washed with PBS and stained with a Live/Dead Staining Kit (InvivoGen, San Diego, CA, USA), anti-CD8-FITC (BD Bioscience), and anti-CD4-BV421 (BD Biosciences) for 20 min at 23°C to stain T cell surface markers. Cells were fixed and permeabilized using a Cytofix/Cytoperm kit (BD Bioscience) for 20 min at 4°C, and then intracellular cytokines were stained with anti-IFN-γ-PE (BD Biosciences), anti-IL-5-APC (BD Bioscience), and anti-IL-17A-PE-Cy7 (BD Bioscience) for 20 min at 23°C. After staining, the cells were analyzed using a MACS Quant flow cytometer (Miltenyi Biotec, San Diego, CA, USA) and FlowJo software (TreeStar, Ashland, OR, USA).

### Cytokine ELISA

Splenocyte culture supernatants prepared as described above were collected, and the levels of IFN-γ, IL-5, IL-17A, TNF-α, IL-10, and TGF-β were measured using an ELISA kit (eBioscience Inc., San Diego, CA, USA).

### Adoptive Transfer of CD4^+^ or CD8^+^ T Cells

Mouse spleen cell suspensions were prepared by passing spleen specimens through a nylon cell strainer (BD Biosciences), and red blood cells were lysed with FACS Lysing Solution (BD Biosciences). Splenic CD4^+^ and CD8^+^ T cells were separated using Miltenyi MACS microbeads conjugated with anti-CD4 and anti-CD8 monoclonal antibodies (Miltenyi Biotec) and a MACS LS column (Miltenyi Biotec). Isolated CD4^+^ or CD8^+^ T cells (5 × 10^5^ cells/mouse) were administered i.p. to naïve C57BL/6 mice (n = 7). After 12 h, mice were challenged i.p. with *S.* Gallinarum 07Q015 (5 × 10^5^ CFU/mouse) and mouse survival was monitored for 12 d.

### Statistical Analysis

Data are expressed as the mean ± standard deviation (SD). Data in the bar and dot graphs between groups were compared using an unpaired Student’s *t*-test for normal data distribution or the Mann–Whitney non-parametric test for abnormal data distribution using GraphPad Prism (version 7.0; GraphPad Software, Inc., La Jolla, CA, USA). The survival of mice was determined using Kaplan–Meier survival analysis, and the significance of the difference was analyzed using a log-rank test with GraphPad Prism software. P < 0.05 was considered statistically significant.

## Results

### Preparation of an Inactivated *S.* Gallinarum Vaccine

**Figure 1:**
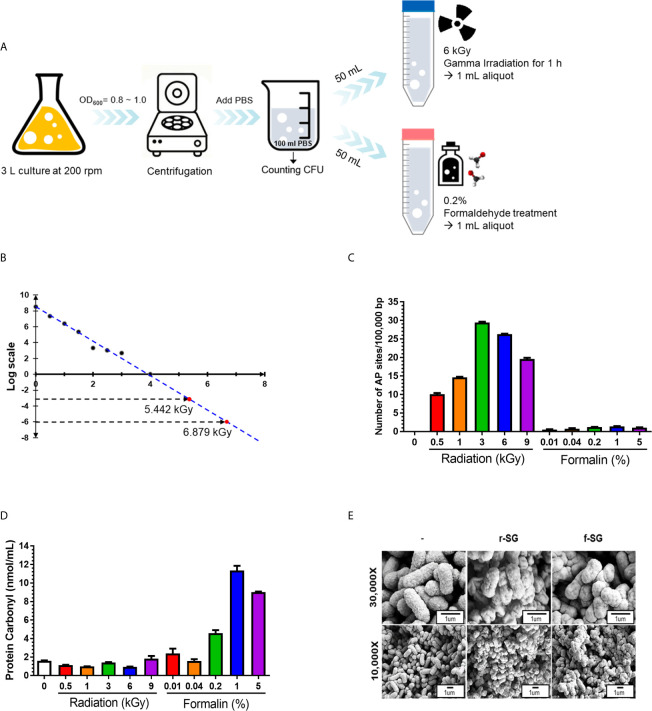
Production and characterization of inactivated SG vaccines. **(A)** Schematic procedure for r-SG and f-SG vaccine production. **(B)** Radiation-inactivated vaccine was produced according to the ‘Sterility Assurance Level (SAL).’ SG was harvested and re-suspended in PBS and irradiated with the indicated dose of gamma radiation (^60^Co gamma-ray source) and plated on LB agar to count the number of surviving bacteria. No SG was detected in samples irradiated with doses of gamma radiation >3 kGy. **(C)** Measurement of single strand chromosomal DNA breaks and **(D)** bacterial protein carbonylation after radiation- or formalin-inactivation. **(E)** The surface structure of SG, r-SG, and f-SG visualized by scanning electron microscopy.

The formalin inactivation method used in this study followed the bacterial vaccine manufacturing protocol of the Korean Animal and Plant Quarantine, in which SG (10^8^–10^9^ CFU/mL) was treated with 0.2% formaldehyde solution in PBS for 2 h at 37°C. For radiation-inactivated SG vaccines, a value between −10^3^ and −10^6^ was applied according to the ‘Sterility Assurance Level (SAL)’ used in the manufacture of sterile viruses (24, 25). To measure the SAL value, harvested SG (10^8^–10^9^ CFU/mL) in PBS were irradiated with gamma rays at the indicated dose for 1 h, and serially diluted samples were plated onto blood agar plate (BAP) (**Figure 1A**). No bacteria were detected on BAP at 3~4 kGy. SAL values of –10^3^ and –10^6^ were calculated as assessed by linear regression performed with all viable count data, indicating that SAL values of –10^3^ and –10^6^ were obtained with doses of 5.442 kGy and 6.879 kGy, respectively. Thus, 6 kGy was chosen as the radiation inactivation dose for SG (**Figure 1B**). After inactivating SG with either formalin or radiation, 1 mL inactivated bacteria was inoculated into 9 mL LB and incubated for 7 d to confirm complete inactivation.

When radiation passes through a cell, it induces an ionization process that produces free radicals *via* the radiolysis of water (26–28), causing DNA damage. To determine whether the radiation inactivation method successfully induced SG DNA damage, Aldehyde Reactive Probe (ARP) was used to examine the DNA single-strand break rate of the SG chromosome (29). As shown in **Figure 1C**, SG DNA damage extensively increased with increased irradiation dose, and approximately (23.252 ± 0.253) sites/100,000 bp damage was detected at 6 kGy. In contrast, only (1.216 ± 0.102) sites/100,000 bp were detected with f-SG, which was comparable to live SG ([0.058 ± 0.001] sites/100,000 bp).

Since formalin- and radiation-induced reactive oxygen species (ROS) can induce protein antigen damage by carbonylation, which can trigger a Th2-biased immune response, the degree of carbonylation was analyzed by a 2,4-dinitrophenylhydrazine (DNPH) immunoassay (30). As shown in **Figure 1D**, r-SG (6 kGy inactivating does) did not show a significant increase in carbonylation ([0.955 ± 0.032] nmol/mL) compared to live SG ([1.602 ± 0.048] nmol/mL). However, 0.2% f-SG exhibited 4.8-fold higher carbonylation ([4.591 ± 0.450] nmol/mL) compared to r-SG (**Figure 1D**). To directly visualize the extracellular structures of live SG, r-SG, and f-SG, scanning electron microscopy (SEM) was performed. As shown in **Figure 1E**, there were no substantial differences in the extracellular structure between the groups.

### Vaccination With r-SG Causes a Stronger Humoral Immune Response

**Figure 2:**
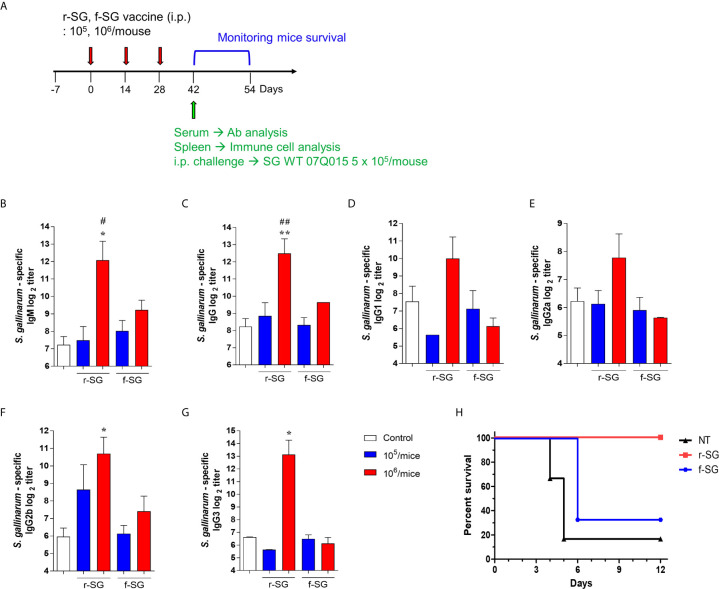
Analysis of humoral immune responses induced by immunization of r-SG or f-SG. Mice (n = 5 per group) were immunized i.p. with either 1 × 10^5^ CFU or 1 × 10^6^ CFU of r-SG or f-SG three times at two week intervals and sera were collected two weeks after the last vaccination. **(A)** Schematic overview of mice study design. SG-specific **(B)** IgM and **(C)** IgG and IgG subclasses **(D)** IgG1, **(E)** IgG2a, **(F)** IgG2b, and **(G)** IgG3 in sera were measured by ELISA. **(H)** Immunized mice (n = 6 per group) were infected i.p. with 5 × 10^5^ CFU SG WT strain and survival was monitored for 12 d. *P < 0.05, **P < 0.01 compared to PBS group. ^#^P < 0.05, ^##^P < 0.01 compared to f-SG vaccinated group.

Because previous studies indicated that high carbonylation induced by formalin treatment decreased antigen-specific humoral immune responses (31, 32), we compared r-SG with f-SG *in vivo*. Mice (n = 5 per group) were immunized i.p. with either 10^5^ or 10^6^ CFU of either r-SG or f-SG vaccine three times at two week intervals, then the SG-specific serum antibody titer was measured two weeks after the last immunization (**Figure 2A**). SG-specific IgM and IgG were increased in a dose-dependent manner by immunizing with either r-SG or f-SG. However, the group immunized with 10^6^ CFU r-SG showed significant enhancement in SG-specific IgM (4,320 ± 2,170) and IgG (5,760 ± 2,061) compared to the f-SG vaccination group (600 ± 126 for IgM, 800 ± 0 for IgG) (**Figures 2B, C**). To determine whether this SG-specific IgG enhancement by r-SG vaccination was due to a Th1- or Th2-biased IgG response, we analyzed the levels of IgG subclasses. As shown in **Figures 2D, E**, both IgG1 (Th2; 1,020 ± 618) and IgG2a (Th1; 220 ± 78) were modestly increased in the r-SG group, but not significantly when compared to the PBS (188 ± 77 for IgG1, 75 ± 14 for IgG2a) and f-SG (70 ± 12 for IgG1, 50 ± 0 for IgG2a) groups. Surprisingly, T-independent antibodies IgG2b (r-SG: 1,160 ± 682, f-SG: 170 ± 62) and IgG3 (r-SG: 8,990 ± 4,775, f-SG: 70 ± 12) were found to be significantly increased in the r-SG group (**Figures 2F, G**).

To confirm whether the SG-specific immune responses induced by the r-SG vaccine could provide protection against SG, immunized mice (n = 6 per group) were i.p. challenged with 5 × 10^5^ CFU WT SG two weeks after the last immunization. While only 16% of PBS-immunized mice, and 33% of f-SG-immunized mice, survived by 6 d post-injection, 100% of r-SG-immunized mice survived at 12 d post-injection. This suggested that r-SG might induce more potent humoral and protective immune responses than f-SG (**Figure 2H**).

### Higher Functional Antibody Responses Induced by r-SG

**Figure 3:**
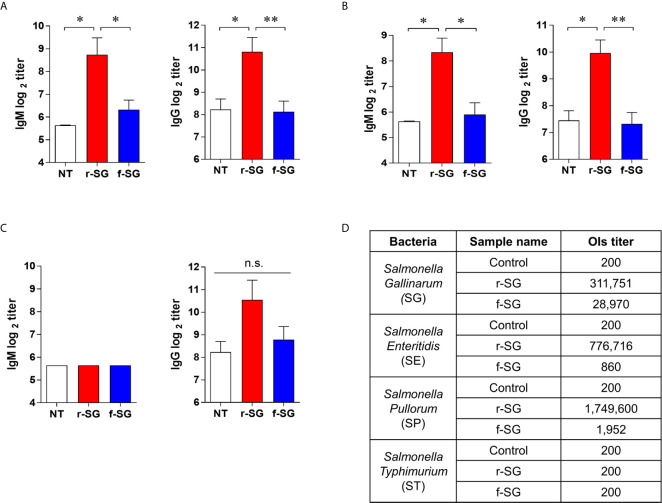
Homogeneous antibody responses by r-SG against Group D Salmonella. Mice (n = 5 per group) were immunized i.p. with 1 × 10^6^ CFU of r-SG or f-SG three times at two week intervals and sera were collected two weeks after the last vaccination. **(A)** SE-specific IgM and IgG, **(B)** SP-specific IgM and IgG, and **(C)** ST-specific IgM and IgG in sera were measured by ELISA. **(D)** Summary of opsonization indices for r-SG and f-SG vaccines against serogroup D (SG, SE, and SP) and serogroup B (ST) Salmonella. *P < 0.05, **P < 0.01, n.s., not significant, compared to unvaccinated mice.

IgG2b and IgG3 have long been considered the key subclasses produced in response to carbohydrates and other T-independent antigens, such as LPS and pneumococcal polysaccharide. Thus, we next sought to confirm that the higher T-independent humoral immune response observed was due to the immune response to SG serogroup D carbohydrate by measuring homogeneous antibody titers of SG-immunized mouse sera against serogroup D (SE, SP) and serogroup B (ST) strains. Sera isolated from mice immunized with 10^6^ CFU of either r-SG or f-SG were used to determine the levels of SE-, SP-, or ST-specific antibody titers by ELISA. As shown in **Figures 3A, B**, serogroup D Salmonella (SE and SP)-specific IgM (r-SG: 425 ± 143, 325 ± 75 f-SG: 80 ± 12, 60 ± 10) and IgG (r-SG: 1,800 ± 503, 325 ± 75 f-SG: 280 ± 48, 60 ± 10) isotype responses increased significantly in the sera of r-SG-immunized mice. As mentioned above, the major subclasses of IgG were IgG2b and IgG3 against SE and SP. In contrast, no significant levels of IgM (r-SG: 50 ± 0, f-SG: 50 ± 0) and IgG (r-SG: 1,500 ± 619, f-SG: 440 ± 97) were detected in serogroup B Salmonella (ST) (**Figure 3C**). Moreover, we found that these serogroup specific antibodies bound only to serogroup D LPS, not serogroup B LPS (**Supplementary Figure 1**). These data indicate that r-SG-induced IgG2b, and IgG3 antibodies were mostly group D-specific. Furthermore, we found that both r-SG and f-SG boosted high group D LPS-specific IgG and IgM, but not group B LPS-specific antibodies.

To demonstrate that the increase in the homogeneous IgG response was not due to non-specific binding to the immobilized antigen, we measured the functional activity of SG-immunized mouse sera using an OPKA, as described previously (33). Live SG, SE, SP, or ST (100–250 CFU) were incubated for 45 min at 37°C with the pooled SG-immunized sera together with baby rabbit complement and differentiated granulocytes (HL60), and the surviving bacteria were counted on LB agar plates. As shown in **Figure 3D**, the opsonic index (OI; 50% killing serum titer) of PBS-immunized sera (control) was 200, which is the OPKA detection limit in all Salmonella groups. Sera immunized with f-SG showed OI of 28,970 to SG, but <2,000 for other serogroups. In contrast, sera immunized with r-SG had extremely high OI titers against group D Salmonella: 311,751 (SG), 776,716 (SE), and 1,749,600 (SP); while OI titers to ST were only 200. These results confirmed that the radiation inactivation method induced a higher serotype-specific humoral immune response than the formalin inactivation.

### Higher SG-Specific Th17 Cell Immunity Induced by r-SG

**Figure 4:**
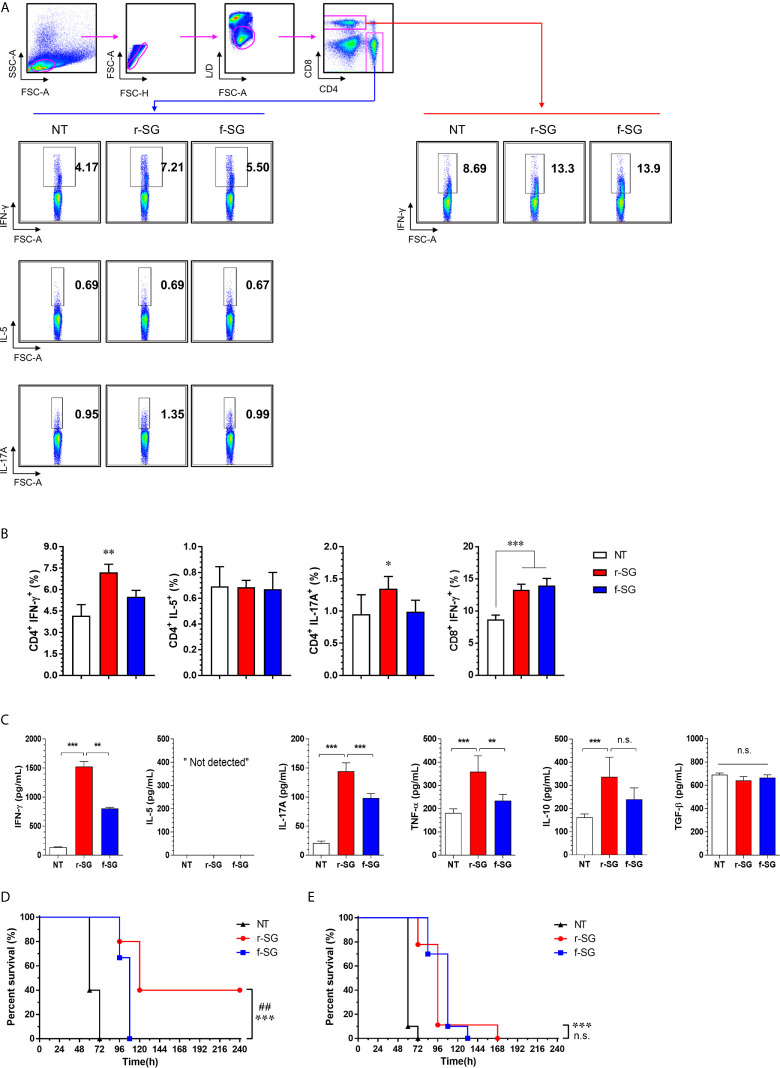
Analysis of SG-specific CD4^+^ and CD8^+^ T-cell responses. Mice (n = 5 per group) were immunized i.p. with 1 × 10^6^ CFU of r-SG or f-SG three times at two week intervals. Spleen cell suspensions were re-stimulated with 10 μg/mL SG lysate for 12 h and SG-specific Th1 (IFN-γ-expressing CD4^+^ T cells), Th2 (IL-5-expressing CD4^+^ T cells), Th17 (IL-17A-expressing CD4^+^ T cells), and activated CD8^+^ T cells (IFN-γ-producing CD8^+^ T cells) were analyzed by intracellular cytokine staining based on the T cell-specific makers (anti-CD4, and anti-CD8). **(A)** Representative plots for Th1, Th2, Th17, and activated CD8^+^ T cells in spleens from PBS-, r-SG-, and f-SG-vaccinated mice. **(B)** Percentages of Th1, Th2, Th17, and activated CD8^+^ T cells in spleens of all vaccinated mice. The mean ± SD shown are representative of three independent experiments. **(C)** Single cell suspensions of splenocytes were treated with 10 μg/mL SG lysate for 72 h, and supernatants were collected for determination of SG-specific cytokines (IFN-γ, IL-5, IL-17A, TNF-α, IL-10, and TGF-b) using ELISA. The mean ± SD shown are representative of two independent experiments. *P < 0.05, **P < 0.01, and ***P < 0.001, n.s., not significant, compared with unvaccinated mice **(D)** Splenic CD4^+^ or **(E)** CD8+ T cells from naïve (n = 7) or r-SG- or f-SG- vaccinated mice were pooled and transferred i.p. to mice. At 12 h after inoculation, mice were challenged i.p. with 5 × 10^5^ CFU SG WT strain. Mouse survival was monitored for 10 d. ***P < 0.001, compared with unvaccinated mice. ^##^P < 0.01, n.s., not significant, compared with f-SG vaccinated mice.

To more accurately confirm the immune-induced response to r-SG, we analyzed cell-mediated immune activity in a population of activated CD4^+^ and CD8^+^ T cells *in vivo*. Mice (n = 5 per group) were immunized i.p. with PBS, r-SG (10^6^ CFU), or f-SG (10^6^ CFU) three times at two week intervals, and single cell suspensions of splenocytes were re-stimulated with 10 µg/mL SG lysate followed by analysis of Th1 (IFN-γ-producing CD4^+^ T cells), Th2 (IL-5-producing CD4^+^ T cells), Th17 (IL-17A-producing CD4^+^ T cells), and activated CD8^+^ T cells (IFN-γ-producing CD8^+^ T cells) by gating (**Figure 4A**). As shown in **Figure 4B**, any group immunized with either r-SG or f-SG showed no significant difference in IL-5^+^CD4^+^ cells compared to the PBS group. Only the r-SG-immunized group showed a significant level of IFN-γ^+^CD4^+^ (r-SG: 7.12 ± 0.56, f-SG: 5.50 ± 0.44; P = 0.005) and IL-17A^+^CD4^+^ cells (r-SG: 1.34 ± 0.19, f-SG: 0.99 ± 0.18; P = 0.03). Both the r-SG and f-SG groups had significant increases in IFN-γ^+^CD8^+^ cells compared to the PBS group, but no differences were found between the r-SG and f-SG groups.

Next, the levels of cytokines secreted from splenocytes in response to SG lysate stimulation were measured by ELISA (**Figure 4C**). Consistent with the above results, significantly higher levels of IFN-γ and IL-17A were detected in the r-SG group compared to the PBS and f-SG groups, but IL-5 production was not detected in any group. In addition, we measured the levels of additional cytokines (IL-10 and TNF-α) associated with Salmonella infections (34, 35). TNF-α was significantly increased (r-SG: 359.1 ± 30.53, f-SG: 234.8 ± 12.3; P = 0.0054), but a modest increase of IL-10 was observed in the r-SG group compared to the f-SG group. Although TGF-β is known to be required for class-switching from IgG3 to IgG2b (36), no significant production of TGF-β was detected in either the r-SG or f-SG groups These data confirm that r-SG could induce higher SG-specific immune responses in the direction of Th1 and Th17, and that a higher IgG2b humoral response was not likely to be in the direction of the TGF-β pathway.

To directly examine whether T cells memorized *via* the r-SG or f-SG vaccine could protect against SG infection, either splenic CD4^+^ or CD8^+^ T cells were isolated from immunized mice and then transferred to naïve mice *via* the i.p. route. Mice were then challenged with a lethal dose of SG (5 × 10^5^ CFU) 16 h after adaptive transfer. As shown in **Figures 4D, E**, the group that received CD4^+^ T cells from PBS- or f-SG-vaccinated mice died within 5 d after injection, whereas the group that received CD4^+^ T cells from r-SG-vaccinated mice showed 40% survival during the monitoring period, which was significant compared to the PBS (P < 0.0001) and f-SG (P = 0.003) groups. In contrast, mice that received CD8^+^ T cells from r-SG- or f-SG-vaccinated donors survived longer than mice in the PBS group, but no significant differences were found between the r-SG and f-SG groups, which correlated with the flow cytometry and ELISA data above. These data suggest that the higher protective immune response induced by the r-SG vaccine could be caused in part by higher activated CD4^+^ T cells, particularly associated with Th1 and Th17 cells.

### Higher Protective Immune Responses by r-SG Vaccination in Chickens

**Figure 5:**
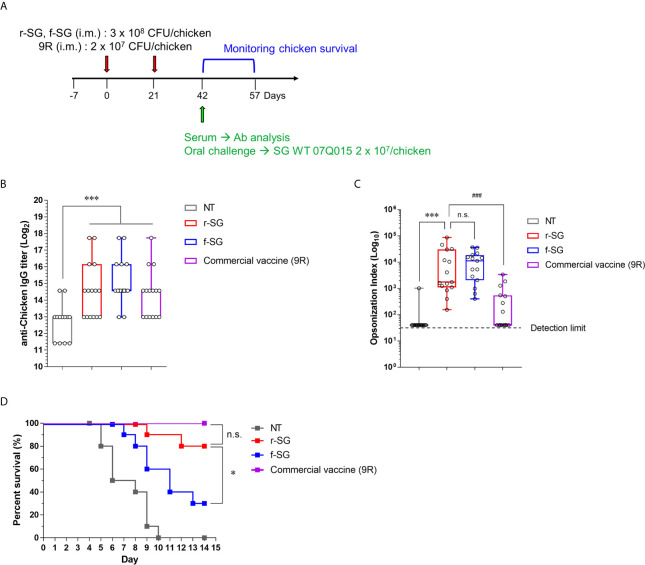
Analysis of humoral immune responses induced by immunization of r-SG or f-SG in chickens. Brown Leghorn chickens (n = 15 per group) were immunized i.m. with the indicated dose of r-SG, f-SG, or live 9R twice at two week intervals. **(A)** Schematic overview of chicken study design. **(B)** A box-and-whisker plot with data points of SG-specific chicken IgG in sera were measured by ELISA and **(C)** Opsonization indices for r-SG, f-SG, and 9R vaccines against *S.* Gallinarum. **(D)** At two weeks after the last vaccination, chickens were orally challenged with 2 x 10^9^ CFU SG WT and survival was monitored for 14 d. *P < 0.05, ***P < 0.001, n.s., not significant, compared with unvaccinated chicken. ^###^P < 0.001, compared with 9R vaccinated chicken.

To compare the efficacy of the vaccine between r-SG and f-SG in chickens, five-week-old female Brown Leghorn chickens were immunized with r-SG (3 × 10^8^ CFU), f-SG (3 × 10^8^ CFU), or commercial 9R live vaccine (2 × 10^7^ CFU) twice at three-week intervals (**Figure 5A**). Sera were collected 14 d after the last vaccination, and serogroup D Salmonella (SG, SE, and SP)-specific IgG were measured using ELISA. As shown in **Figure 5B**, all groups immunized with r-SG, f-SG, or 9R showed a significant increase in Group D-specific IgG antibodies (r-SG: 50,220 ± 18,491, f-SG: 57,780 ± 17,779, 9R: 37,260 ± 14,070) compared to the unvaccinated group, but there was no significant difference among the vaccinated groups. Next, we measured the functional activity of SG-immunized chicken sera using OPKA against live SG. As shown in **Figure 5C**, the opsonic index (OI; 50% killing serum titer) was 105 ± 65 (PBS), 15,376 ± 6,332 (r-SG), 12,318 ± 3,069 (f-SG), and 553 ± 245 (9R) indicating that both f-SG and r-SG induced higher functional SG-specific antibodies than the live 9R vaccine (**Figure 5C**).

Two weeks after the last vaccination, the immunized chickens (n = 15) were orally challenged with 3 × 10^7^ CFU of *S.* Gallinarum 07Q015 (week 6). All PBS-immunized chickens died within 10 d, but 100% (Live/Dead: 15/15) and 80% (Live/Dead: 12/15) of the 9R- and r-SG-immunized chickens, respectively, were alive for more than 14 d post-challenge (**Figure 5D**). In contrast, only 33% (Live/Dead: 5/15) of the f-SG-immunized chickens survived for 14 d after challenge. Taken together, the data indicated that the higher protective immune response of r-SG vaccination was likely due to both the higher humoral immune response and cellular immune response.

## Discussion

Inactivated vaccines have been the most widely used type of vaccine since the 1920s, but due to the recent emergence of new strains and subtypes, it is becoming difficult to sufficiently prevent infectious diseases with inactivated vaccines (37). Instead, live vaccines are being used as alternatives, but their use is extremely limited because of their difficulty in development and high virulence to immunocompromised humans and animals (38). For example, the SG 9R live vaccine against SG has recently been reported as an unpredictable invasive infection (8). Thus, there is an urgent need to develop safe new SG vaccines. In this study, we found that a r-SG vaccine could provide a higher homogeneous protective response against group D Salmonella by activating early humoral responses (IgG2b and IgG3) and Th1/Th17 cell-mediated immunity than f-SG.

Multiple studies have shown that radiation-inactivated vaccines provide better efficacy than conventional vaccines (13). Furuya et al. reported that influenza vaccines inactivated with gamma radiation significantly enhanced the activity of cytotoxic T cells, which could provide heterosubtypic protection against various influenza subtypes, including the H5N1 avian influenza virus (39). Although ultraviolet radiation was used to inactivate Listeria in a study by Brockstedt et al., they reported that this could induce a potent CD8^+^ T cell response to increase cellular immunity (40). However, our study found that the r-SG vaccine activates SG-specific Th1/Th17 cells more than cytotoxic T cells, possibly providing high protective immunity. In fact, we recently demonstrated that radiation-inactivated pneumococcal vaccines provide protection *via* Th17-mediated mucosal immune responses (41). Thus, radiation inactivation methods generally provide stronger protective immune responses, but the type of response induced may differ depending on the type of pathogen. As the molecular mechanism underlying the high immune response of radiation-inactivated vaccines is not yet clear, the key determinants leading to the activation of cytotoxic T cells or Th17 cells should also be studied.

Formaldehyde is an electrophilic aldehyde that induces chemical crosslinking of the N-terminal nucleophilic amino group of cysteine, histidine, lysine, tryptophan, and arginine (42). It is well known that the use of formaldehyde, in the form of the aqueous solution formalin, for vaccine production can cause a variety of immunological issues because it can alter the structure of antigenic epitopes (43, 44) and diminish the production of pathogen-specific and functional antibodies. We reported that formalin inactivation could decrease the humoral immune response by impairing the activity of influenza surface antigens hemagglutinin (HA) and neuraminidase (NA) (45). In this study, we also showed that formalin treatment of SG (f-SG) dramatically increased the carbonylation of SG proteins compared to untreated cells or r-SG. Second, formalin is known to increase unpredictable immune responses, such as antibody-dependent enhancement (ADE) (46). For example, a formalin-inactivated respiratory syncytial virus (RSV) vaccine in naïve infants failed to prevent disease, and 80% of vaccine recipients were hospitalized after encountering circulating RSV due to ADE (47). The issue of the SARS-CoV-2 vaccine inducing ADE following structural modifications of the surface S-protein is also a concern (48). Unlike chemical inactivation, radiation inactivation in cells can be caused by direct or indirect action on DNA (49). Radiation can directly cause DNA single-or double-strand breaks (50), while the indirect action causes ionization of water or organic molecules in the cell to produce free radicals which react with DNA bases, leading to DNA damage (51). Thus, the two actions can function together to cause various mutations and break down DNA to quickly inactivate the pathogen. In contrast, cellular damage might be relatively less compared to DNA damage because the physical process of ionizing radiation takes a very short reaction time (about 10^-15^ to 10^-12^ s) (26, 27, 52). Therefore, the high immunological effect of irradiated vaccines may be due to less damage to surface antigens during vaccine manufacturing.

Another surprising finding in our mouse model was that r-SG induced higher titers of SG-specific IgM, IgG2b, and IgG3, and these antibodies are probably anti-group D antigens. IgG2b and IgG3 antibodies are usually considered to be part of the T-independent response and can be induced at a very early stage of infection (53). A previous study showed that LPS stimulation could induce class switching of IgM to IgG3, and LPS induces high levels of IgG2b and low levels of IgA in the presence of TGF-β (54). However, we did not detect the secretion of TGF-β, and there is another, unknown, mechanism for class switching to IgG2b. In addition, both IgG3 and IgG2b are known to have stronger opsonophagocytosis and bactericidal activities than IgG2a and IgG1 (55), therefore the r-SG vaccine may provide early and robust protective humoral immune responses *via* IgG3 and IgG2b. In contrast, we detected a relatively low amount of SG-specific T-dependent antibodies (IgG1, IgG2a) by r-SG vaccination, even with aluminum hydroxide as an adjuvant. Therefore, there is a strong need to investigate the impact of a specific adjuvant combination in increasing the Th1 response.

Although we have not been able to directly analyze the response of cellular immunity after r-SG vaccination in chickens, the increase in protective immunity is likely to be due to an increase in cellular immunity. The role of Th1- and Th17-mediated immune responses has been reported in many infectious diseases, such as those caused by Candida, Salmonella, and Pneumococcus (56–59). Following Salmonella infection, the Th17 response in the CD4^+^ T cell population shifts to a Th1-biased response, and IL-17A, which is increased by Salmonella infection, stimulates intestinal epithelial cells to enhance the production of antimicrobial proteins and chemokines (60, 61). Thus, the higher protective immune response induced by r-SG might be due to Th17-mediated Th1-biased response. Further analysis of the cellular immune responses should be performed on the spleen of chickens given various doses of r-SG vaccine.

Ionizing radiation primarily damages DNA and consequently, the biological responses depending on the radiation track structure and its energy loss distribution pattern (62). However, because it is difficult to investigate the exact mechanism of ionizing radiation at the atomic level, several modeling and simulation programs, such as Geant4-DNA, have been introduced to predict the effects of cellular oxygenation on the chemical processes involving DNA radicals (63). According to Geant4-DNA simulation, low linear energy transfer radiation can cause 0.04 to 0.010 double-strand breaks/Gy/Mbp, indicating that radiation can penetrate the cell nucleus to induce intracellular oxygenation processes for double-strand breaks in DNA. In contrast, formaldehyde causes external damage to the cell through a diffusion mechanism. Therefore, irradiated microorganisms have the potential to be recognized as living microorganisms in part through the interaction of well-conserved surface antigens with immune cells, which can promote the cell-mediated immune response.

In summary, we are the first to develop a gamma radiation-inactivated vaccine to SG that is safer and more effective than live SG 9R or chemically-inactivated SG vaccines. We found that the r-SG vaccine has the advantage of inducing a higher humoral immune response than the live 9R vaccine and a higher cell-mediated immune response than f-SG. Therefore, the development of vaccines using gamma irradiation is expected to be applicable to various infectious diseases as an effective vaccine manufacturing method capable of inducing an immune response at an intermediate level between inactive and live vaccines.

## Data Availability Statement

The original contributions presented in the study are included in the article/**Supplementary Material**. Further inquiries can be directed to the corresponding authors.

## Ethics Statement

The animal study was reviewed and approved by KAERI-IACUC.

## Author Contributions

HJJ, JH, SHH, HKJ, and JHL were responsible for conceptualization of the study, HJJ, EBB, FC, KBA, YW, and JYM performed the experiments and analyzed the data. HJJ, SHH, JHL, and HSS wrote the manuscript. HSS supervised the work. HSS, JH, and HKJ were responsible for funding acquisition.

## Funding

This work was supported in part by National Research Foundation of Korea grants NRF-2017M2A2A6A02020925, NRF-2018K2A206023828, and NRF-2020M2A206023828 to HSS.

## Conflict of Interest

Authors HKJ and YW were employed by company HONGCHEON CTCVAC Co., Ltd.

The remaining authors declare that the research was conducted in the absence of any commercial or financial relationships that could be construed as a potential conflict of interest.

## Publisher’s Note

All claims expressed in this article are solely those of the authors and do not necessarily represent those of their affiliated organizations, or those of the publisher, the editors and the reviewers. Any product that may be evaluated in this article, or claim that may be made by its manufacturer, is not guaranteed or endorsed by the publisher.

## Supplementary Material

Supplementary Figure 1 Measurement of Serogroup B or D specific LPS antibody responses induced by r-SG. Mice (n = 5 per group) were immunized i.p. with 1 × 10^6^ CFU of r-SG or f-SG three times at two week intervals and sera were collected two weeks after the last vaccination. **(A, B)** Serogroup B or D LPS specific antibody levels were measured by ELISA. Serogroup B and D-specific IgM **(A)** and Serogroup B and D-specific IgG **(B)**. *P < 0.05, **P < 0.01, ***P < 0.001, compared to unvaccinated mice or f-SG vaccinated group.
