# Supplementary figures and images for: Radiation-Inactivated S. gallinarum Vaccine Provides a High Protective Immune Response by Activating Both Humoral and Cellular Immunity
Source: Front Immunol. 2021 Aug 16;12:717556. doi: 10.3389/fimmu.2021.717556 (PMC8415480; doi:10.3389/fimmu.2021.717556)

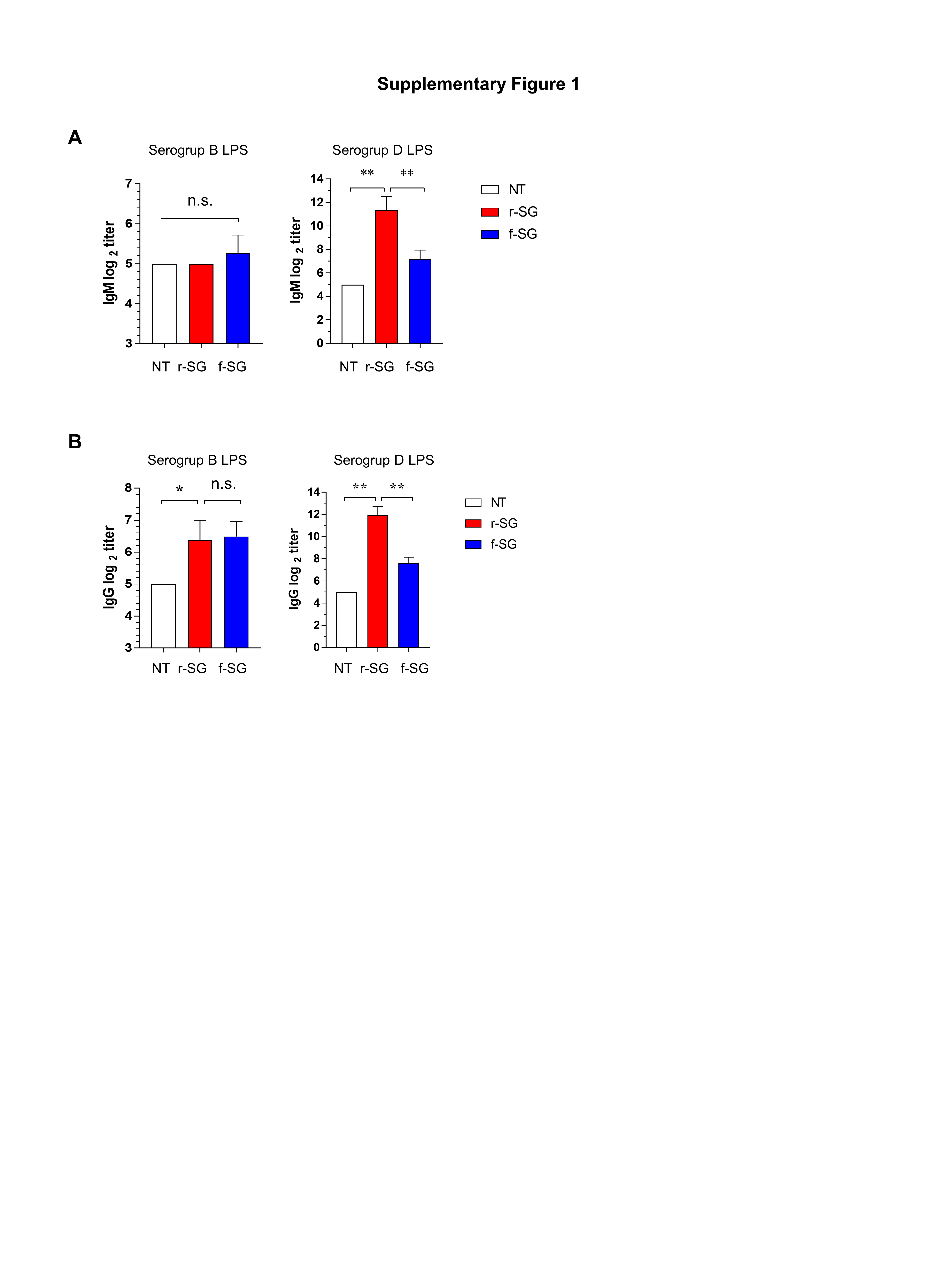

Supplement: Supplementary Figure 1 — Measurement of Serogroup B or D specific LPS antibody responses induced by r-SG. Mice (n = 5 per group) were immunized i.p. with 1 × 10^6^ CFU of r-SG or f-SG three times at two week intervals and sera were collected two weeks after the last vaccination. (A, B) Serogroup B or D LPS specific antibody levels were measured by ELISA. Serogroup B and D-specific IgM (A) and Serogroup B and D-specific IgG (B). *P < 0.05, **P < 0.01, ***P < 0.001, compared to unvaccinated mice or f-SG vaccinated group. [file Image_1.tif]
